# Prevalence and antimicrobial resistance of shiga toxin-producing *Escherichia coli* and enteropathogenic *Escherichia coli* isolated from patients with acute diarrhea

**Published:** 2018-06

**Authors:** Saeid Bouzari, Elham Farhang, Seyed Mostafa Hosseini, Mohammad Yousef Alikhani

**Affiliations:** 1 Molecular Biology Department, Pasteur Institute of Iran, Tehran, Iran; 2 Microbiology Department & Brucellosis Research Center, Hamadan University of Medical Sciences, Hamadan, Iran

**Keywords:** Diarrhea, *Enteropathogenic Escherichia coli*, *Enterohaemorrhagic Escherichia coli*, *Shiga toxin-producing Escherichia coli*

## Abstract

**Background and Objectives:**

Diarrheal disease is still a major health problem in developing countries, where it is considered as one of the leading causes of morbidity and mortality especially in children. *Escherichia coli* is one of the important enteropathogenic bacteria which causes diarrhea in people. The aim of this study was to investigate the prevalence and antimicrobial resistance of shiga toxin–producing *E. coli* (STEC), Enterohaemorrhagic *E. coli* (EHEC), and Enteropathogenic *E. coli* (EPEC) in fecal samples collected from patients with acute diarrhea in a number of Iranian provinces.

**Materials and Methods:**

A total of 102 strains of *E. coli* were isolated from fecal samples collected from patients with acute diarrhea using microbiological phenotypic tests. The antibiotic susceptibility pattern of all isolates was determined by the disk agar diffusion (DAD) method. The presence of *eae, bfp, stx1, sts2* and *EAF* genes in the isolates was investigated by PCR. The results were analyzed by SPSS; version 17.0 software.

**Results:**

Out of 102 *E. coli* isolates screened for specific genes, 52 strains of *E. coli* were identified to harbor STEC 26 (50%), EPEC 13 (25%) and EHEC 13 (25%). Greatest resistance was observed to amoxicillin and ampicillin 40 (76.9%), and most sensitivity to imipenem 52 (100%) and gentamicin 40 (76.9%). We also found that 80.77% of diarrheic *E. coli* isolates were multidrug resistant (MDR).

**Conclusion:**

The results showed that *E. coli* is one of the major causes of diarrhea and is highly resistant to commonly used antibiotics; therefore, officials must pay great attention to this issue in order to increase the health of the community.

## INTRODUCTION

Diarrheal disease is still a major health problem and is one of the most common causes of morbidity and mortality among infants and young children, especially in developing countries, accounting for around 2 million deaths annually (1, 2). In the past decades, diarrhea was one of the major causes of infant deaths in Iran (3). Among the bacterial pathogens, diarrheagenic *Escherichia coli* (DEC) is one of the important etiological agents of diarrhea (4). Strains of diarrheagenic *E. coli*, which cause diarrhea in humans, can be classified into at least seven different categories on the basis of their specific virulence properties, distinct epidemiology, and clinical features: Enterotoxigenic *E. coli* (ETEC), Enteroinvasive *E. coli* (EIEC), Enteroaggregative *E. coli* (EAEC), Diffuse-Adhering *E. coli* (DAEC), Cytolethal distending toxin-producing *E. coli*, Enteropathogenic *E. coli* (EPEC), and Enterohaemorrhagic *E. coli* (EHEC) (4, 5). The epidemiological significance of each DEC category in childhood diarrhea varies with the geographical area. As expected, it has become obvious that there are important regional differences in prevalence and other epidemiological features of these pathogens (2, 6).

EPEC was the first pathovar of *E. coli* to be recognized as a diarrheal pathogen (7, 8), and it plays an important role as a causative agent of infantile diarrhea among children in developing countries (9). These strains harbor both the bundle-forming pilus gene (*bfpA*) and Intimin, which is encoded by the chromosomal *eae* gene, which possess the ability to form A/E lesions on intestinal cells and do not contain shiga toxin encoding genes (2).

EPEC and STEC (Shiga toxin–producing *E. coli*) are distinguished by presence of the shiga toxin encoding genes (*stx1* and *stx2*), being present only in STEC (4). The term EHEC is used to denote only the subset of *stx* positive strains that also contain Intimin (*eae* gene), which is part of a pathogenicity island termed the locus for enterocyte effacement (LEE) (10). However, there are LEE-negative STEC strains that are associated with disease (11). To identify DEC strains correctly, these organisms must be differentiated from non-pathogenic members of the normal flora by PCR to identify presence of specific virulence genes, which are absent in nonpathogenic strains (12).

Due to the importance of epidemiological scientific research into the prevalence and occurrence of outbreaks, determination of resistance to antibiotics is very important in bacterial isolates. Further, regular surveillance of antibiotic resistance provides information for antibiotic therapy and resistance control (12). In this study, we determined the prevalence of STEC and EPEC from acute diarrheagenic fecal samples in randomly-selected populations in Iran using culture and PCR to detect presence of *eaeA, stx1, stx2, bfp* and *EAF* genes and determined antimicrobial resistance profiles of the isolates.

## MATERIALS AND METHODS

### Definitions.

Diarrhea was defined as at least three loose stools in 24 h, any number of watery stools, or one or two loose stools in 24 h accompanied by at least one of the following symptoms: nausea, vomiting, abdominal cramps or fever of 38°C. Acute diarrhea was defined as diarrhea that lasted 14 days or less at the time of presentation. Isolates from children with persistent diarrhea were not included in this study. Persistent diarrhea was defined as diarrhea which lasted for more than 14 days at presentation. When diarrhea was present intermittently, it was considered persistent when diarrhea occurred during at least six days in a two-week period (13).

### Sample collections and identification of isolates.

The present study included 102 *E. coli* strains isolated from 158 stool samples collected from patients (7 months to 90 years) with diarrhea who were referred to different hospitals in the north and northwest and western regions of Iran between 2012 and 2013. These isolates were stored on solid Luria-Bertani (LB) (1% tryptone, 0.5% yeast extract, 0.5% NaCl) medium at the Molecular Biology Department of Pasteur Institute of Iran.

At the time of this study in April 2014, a loopful of the LB agar was inoculated on LB broth and incubated at 37°C with shaking for 24 hrs. Then, it was inoculated on MacConkey (Merck, Germany) agar plates and incubated at 37°C for 24 hrs. Subsequently, *E. coli*-like colonies were subjected to different standard biochemical tests, including sugar fermentation, Simmons citrate agar, indole production, Motility, Methyl-Red, and Voges-Proskauer (IMVIC) methods described by Cowan (7). A sweep of five *E. coli* colonies on MacConkey agar were inoculated in LB broth and incubated overnight at 37°C and then stoked at −20°C until used.

### Molecular diagnosis.

*E. coli* genomic DNA was extracted using a DNA extraction kit (Gene JET Plasmid Miniprep., Fermentas Company, Lithuania) according to manufacturer′s instructions. *E. coli* colonies were tested by PCR for the virulence genes. The primers (Table 1) were selected to detect five different virulence genes (*stx1, stx2, eaeA, bfp* and *EAF*) simultaneously in a single reaction. EPEC and EHEC were distinguished by presence of the Shiga toxin-encoding (*stx1* and/or *stx2*) genes, which are present only in EHEC.

**Table 1.** Primer sequences used for the polymerase chain reaction assay in this study

**Name**	**Sequence (5′ to 3′)**	**Size (bp)**
*eaeA*	F CAGGTCGTCGTGTCTGCTAAA	570
*eaeA*	R TCAGCGTGGTTGGATCAACCT	
*stx1*	F CGATGTTACGGTTTGTTACTGTGACAGC	244
*stx1*	R AATGCCACGCTTCCCAGAATTG	
*stx2*	F GTTTTGACCATCTTCGTCTGATTATTGAG	324
*stx2*	R AGCGTAAGGCTTCTGCTGTGAC	
*bfp*	F GACACCTCATTGCTGAAGTCG	910
*bfp*	R CCAGAACACCTCCGTTATGC	
*EAF*	F CAGGGTAAAAGAAAGATGATAA	397
*EAF*	R TATGGGGACCATGTATTATCA	

Amplification was performed in a reaction mixture with a total volume of 25 μL and using 12.5 μL PCR Master Mix 2x, manufactured by FermentasCompany (Lithuania) containing 400 mM deoxy-nucleoside triphosphates, 4 mM MgCl2, 0.05 U/μL Taq DNA polymerase, 5.5 μL sterile Double distilled water, 1 μL from each primers, and 5 μL DNA template. Cycling parameter was used as follows: 95°C for 5 min to initially denature the DNA, then 30 cycles of 1 min at 94°C, 1 min at 50°C, 1 min at 72°C, and finally single prolonged extension at 72°C for 10 min for *eaeA, stx1* and *stx2* genes, and 3 min at 95°C, then 35 cycles of 1 min at 94°C, 45 sec at 55°C, 45 sec at 72°C, and finally a single prolonged extension at 72°C for 10 min for *bfp* and *EAF* genes. A negative control lacking a DNA template and/or *E. coli* K-12 was included in each experiment to exclude the possibility of reagent contamination. The *E. coli* strain used as positive control in the PCR test included *E. coli* ATCC E234864 (*eaeA, bfp a*nd *EAF* positive) and *E. coli* ATCC O157:H7 (*eaeA, stx1* and *stx2* positive) (7). The amplified product was visualized by gel electrophoresis in 1.5% agarose gel containing ethidium bromide for 45 min at 100 V and then visualized under UV light.

### Antimicrobial susceptibility testing.

Antimicrobial susceptibilities of the isolates that yielded positive results in the PCR assay were determined on Mueller-Hinton agar (Merck, Germany) by the Kirby-Bauer method, according to the Clinical and Laboratory Standard Institute (CLSI) protocol (14). Twelve commercial antibacterial discs (PadtanTeb, Iran) from different classes, which are generally used in medical diagnostic laboratories in Iran, were employed. The discs included nalidixic acid (NA, 30 μg), gentamicin (G, 10 μg), ampicillin (AM, 10 μg), amoxicillin/clavulanate (AMC, 30 μg), amoxicillin (AMX, 25 μg), trimethoprim-sulfamethoxazole (STX, 25 μg), tetracycline (TE, 30 μg), ceftriaxone (CRO, 30 μg), cefixime (CFM, 5 μg), ciprofloxacin (CP, 5 μg), cephalothin (CF, 30 μg), and imipenem (IPM, 10 μg).

### Statistical analysis.

The data were analyzed with SSPS version 17.0 software (SPSS). The χ^2^ test was used to determine the statistical significance of the data. A p value of ≤ 0.05 was considered as significant.

## RESULTS

Out of 102 *E. coli* isolates (five colony from each stool sample; 510 colonies) which were identified by biochemical tests, a total of 52 isolates were identified as diarrheagenic *E. coli* by PCR: 13 (25%) EPEC (*eaeA,* and/or *bfp* and/or *EAF* positive), 26 (50%) STEC (*stx1* and/or *stx2*, positive) and 13 (25%) EHEC (*stx1* and/or *stx2* and *eaeA* positive). Among 26 STEC isolates, 15 (57.7%) strains were *stx1*+, 3 (11.5%) isolates were *stx2*+, and 8 (30.8%) isolates were positive for stx1 and *stx2*. The results of our study showed that among 13 EHEC isolates, 8 (61.54%) strains were *eaeA*+ and *stx1*+, 3 (23.08%) isolates were *eaeA*+, *stx1*+, *stx2*+, and 2 (15.38%) isolates were *eaeA*+, *stx2*+. The results showed that among 13 EPEC isolates, 11 (84.62%) strains were *eaeA*+, and 2 (15.38%) isolates were *eaeA*+ and *bfp*+.

Totally, 20 (55.5%) diarrheagenic *E. coli* were isolated from 36 children and 32 (48.4%) of 66 adult with acute diarrhea. The sex distribution was 33 (56%) male and 19 (44%) female. The most prevalent pathotype in the diarrheal patients was STEC (50%) isolates, followed by EHEC (25%) and EPEC (25%).

The results showed that EPEC has the highest incidence in the autumn (11.53), followed by EHEC in the summer (15.38), and STEC in the winter (23.07%). The isolates of *E. coli* were detected in all age groups, and there was no statistical significant difference between the frequency of isolation, age and sex.

### Antimicrobial susceptibility testing.

The results of antimicrobial susceptibility testing of 52 *E. coli* strains isolated from diarrheal patients to 12 antibiotics are shown in Table 2. The most prevalent resistance profiles were ampicillin and amoxicillin (40 isolates, 76.9%) followed by cephalothin (36 isolates, 69.2%). Imipenem was found to be the most effective antibiotic with a susceptible rate of 100% (52 isolates, 100%), followed by gentamicin (40 isolates, 76.9%). Of the 52 strains tested, 42 (80.77%) were multidrug resistant (resistant to more than six antimicrobial drugs).

**Table 2.** Results of antimicrobial resistance of *E. coli* isolates using disk diffusion method.

**Antimicrobial agents**	**Resistant No (%)**			

	**EHEC**	**STEC**	**EPEC**	**Total**
Nalidixic Acid	11 (84.61)	16 (61.53)	6 (46.15)	33 (63.5)
Gentamicin	2 (15.38)	6 (23.07)	1 (7.69)	10 (19.2)
Ampicillin	11 (84.61)	21 (80.76)	8 (61.53)	40 (76.9)
Amoxi-Clav	4 (30.76)	7 (26.92)	1 (7.69)	13 (25)
Amoxicillin	11 (84.61)	20 (76.92)	9 (69.23)	40 (76.9)
Co-trimoxazole	8 (61.53)	6 (23.07)	5 (38.46)	32 (61.5)
Tetracycline	10 (76.92)	18 (69.23)	(46.15)6	34 (65.4)
Ceftriaxone	4 (30.76)	5 (19.23)	2 (15.38)	1 (21.2)
Cefixime	6 (46.15)	7 (26.92)	3 (23.07)	14 (26.9)
Ciprofloxacin	7 (53.84)	7 (26.92)	4 (30.76)	18 (34.6)
Cephalothin	12 (92.30)	18 (69.23)	6 (46.15)	36 (69.2)
Imipenem	0 (0)	0 (0)	0 (0)	0 (0)

## DISCUSSION

Studies in Iran showed that DEC, such as EPEC and STEC strains, are among the most prevalent causative agents in acute diarrhea, particularly in children (15, 16). In our research blood was reported in all feces of patients with the EHEC pathotype, but none of them belonged to the O157:H7 serotype. These data reconfirmed geographical variation and showed absence of the O157:H7 serotype among STEC isolates in our areas (15). Similar results were found in France and in Switzerland (17, 18). Non-O157 STEC may also play a more important role in disease compared to STEC O157:H7 as shown in Argentina, Australia, Chile and South Africa (19–21). In Canada, United States, Japan, England and Scotland, in contrast, prevalence of non-O157 is very low (22). In our study, the highest rate of EHEC (30.77%) isolation was detected in Gilan province, north of Iran, with a higher incidence in summer (61.54%). Some studies have suggested that there is an interesting phenomenon in developing countries in which EHEC is much less frequently isolated than other diarrheagenic *E. coli* (16, 23).

In the present study, the highest rate of diarrhea causing *E. coli* belonged to STEC (50%) isolates. The STEC isolates had higher incidence in Gilan province (30.7%) in the winter season. The least frequency (3.8%) belonged to Hamadan, Zanjan (west of Iran), and East Azerbaijan provinces (northwest of Iran). Our finding show that STEC strains were detected significantly more (76.9%) in adult man with diarrhea. Vilchez et al. identified a few EHEC strains from children with diarrhea, therefore, our findings were in concurrence with the low prevalence of EHEC infection in developing countries (24). Some studies have suggested that there is an interesting phenomenon in developing countries in which EHEC is isolated much less frequently than other DEC strains such as ETEC or EPEC (25). In the present study, STEC was found in 23.08% of diarrheic children. Pourakbari et al. and Jafari et al. reported that prevalence of STEC strains in children with diarrhea in Tehran, Iran, was 17% and 18.9%, respectively (13, 26). In another report, STEC strains were isolated in 15.5% of children with diarrhea in Tehran (27). Our findings are approximately similar to reports from Tehran. Also, outbreaks and sporadic cases of EHEC have been reported in developed countries such as North America, Japan, Europe and even Australia (28).

Our results showed that the prevalence of STEC strains was higher than EPEC strains, like another report in Iran (29, 30). EPEC strains still remain a major cause of mortality in infants in developing countries (19, 31). In our study, none of the *E. coli* isolates carried the *EAF* gene, thus, we did not find any typical EPEC strains. Generally, EPEC divided into two types; typical and atypical. Atypical EPEC contain the LEE but do not contain the EAF plasmid. In industrialized countries, atypical EPEC are more frequently isolated from diarrheal cases than are typical EPEC that contain the EAF plasmid although typical EPEC dominate in developing countries (32). The results of our study revealed that all the EPEC strains isolated in the diarrhea patients were atypical EPEC (i.e. *eaeA*+ and *EAF*−), in accordance with recent findings in developing and developed countries which show increased isolation of atypical EPEC (11, 33).

We discovered higher incidence of EPEC in East Azerbaijan province (30.7%), whereas we did not see any EPEC isolate in Kurdistan province, west of Iran (0.0%). In the present study, EPEC were detected in 53.9% of diarrheic children, despite some previous studies showing higher prevalence of STEC in Iran (34). Similar to this study, Alikhani et al. reported high prevalence of EPEC compared with other pathogenic *E. coli* strains in children with diarrhea in Iran (15). In Brazil, EPEC was more frequent than STEC (35). The results of the present study and most previous research suggest that geographical area and time of sampling are the most important criteria in epidemiology of diarrhea in children in developing countries. We demonstrated high incidence of EPEC isolates in autumn despite findings of Momtaz et al. which showed that the higher incidence of EPEC strains is in the summer (36).

The antimicrobial resistance results for the DEC strains are shown in Table 2. The high incidence of antibiotic-resistant isolates of DEC may be due to the widespread use of antibiotics. Transfer of resistance genes that may occur between species could lead to construction of diverse resistance to usual antibiotics. These transfers have effectively changed the ecological and pathogenic character of bacterial species (37). In our study, the high incidence of antibiotic-resistant isolates of EHEC was seen to cephalothin (92.30%). Some results showed high resistance rates against the commonly used antimicrobial agents; ampicillin, amoxicillin, chloramphenicol, tetracycline, cotrimoxazole and nalidixic acid (38, 39). Further search into the drug sensitivity pattern of our isolates demonstrated a good response to imipenem (100%), gentamicin (76.9%), cefixime (73.1%) and ceftriaxone (71.2%) but resistance to ampicillin, and amoxicillin (76.9%), cephalothin (69.2), and tetracycline (65.4%) was noticeable. Our findings are in agreement with reports in Iran indicating DEC and Shigella isolates are resistant to trimethoprim-sulfamethoxazole, and tetracycline (13, 40). In addition, a report from Iran cited by the World Health Organization indicates that sulfamethoxazole-trimethoprim, tetracycline and chloramphenicol were the least effective antibiotics for treatment of DEC isolates (41). We also found that 80.8% of diarrheic *E. coli* isolates were multidrug resistant (MDR), which was considerably high. The incidence of diarrhea due to MDR *E. coli* has increased in developing countries in the last decade (42, 43). At present, because of the increased frequency of MDR DEC, fluoroquinolones are considered as first-line drugs for treatment of diarrhea. Indeed, several studies have documented the emergence and spread of fluoroquinolone resistant enteric pathogens (44) and thus, monitoring of drug susceptibility of DEC seems to be a critical issue in Iran.

In conclusion, this study reveals that DEC strains contribute to the burden of infant to adult diarrheal diseases, and STEC is the most commonly identified DEC strain in our study. To stop the increasing prevalence rate of MDR DEC, the indiscriminate use of antibiotics needs to be avoided and the guidelines for proper use of antibiotics for treatment of diarrhea in this region needs to be established.
